# Clinical, Electrodiagnostic, Sonographic, and Radiological Findings of Hirayama Disease: A Report of Two Cases and a Review of the Literature

**DOI:** 10.7759/cureus.73914

**Published:** 2024-11-18

**Authors:** Lisa B Shields, Vasudeva G Iyer, Yi Ping Zhang, Christopher B Shields

**Affiliations:** 1 Norton Neuroscience Institute, Norton Healthcare, Louisville, USA; 2 Clinical Neurophysiology, Neurodiagnostic Center of Louisville, Louisville, USA

**Keywords:** benign focal amyotrophy, electrodiagnostic studies, fasciculations, hirayama disease, neurology, polyminimyoclonus, ultrasound

## Abstract

Hirayama disease (HD) is a rare disorder characterized by insidious asymmetric neurogenic atrophy primarily involving the upper extremities. HD most commonly affects adolescent males and has a favorable prognosis for arrest of progression. Electrodiagnostic (EDX) studies show chronic denervation changes in the distal upper extremity muscles. A cervical spine MRI in neck flexion may reveal compression of the cervical ventral spinal cord. We report clinical, EDX, and MRI findings in two cases of HD. The first case involved a 15-year-old male with a six-month history of progressive weakness, wasting, and tremulous movements of the left hand without pain or paresthesia. A physical exam revealed marked wasting of the left intrinsic hand muscles with polyminimyoclonus. Needle electromyography (EMG) revealed fasciculations (simultaneous with the polyminimyoclonus) while at rest and recruitment of 1-2 large polyphasic units in the left C8, T1, and, to a lesser extent, the C7 distribution. A T2-weighted flexion cervical spine MRI revealed narrowing and anterior displacement of the posterior dura of the cervical cord, leading to cord compression at C5-6 and C6-7. At last contact 11 years following symptom initiation, the patient continued to complain of profound weakness of the left hand without pain or numbness. The second case involved a 17-year-old male who was found to have wasting of the intrinsic muscles of the non-dominant left hand on a routine physical examination. The left first dorsal interosseus, abductor digiti minimi, abductor pollicis brevis, and extensor pollicis longus muscles were found to be clinically weak. Polyminimyoclonus involving all fingers of the left hand was also observed. A needle EMG demonstrated fasciculations with large amplitudes and wide duration motor unit potentials in the left C8, T1, and, to a lesser extent, C7 distribution. An ultrasound study showed frequent fasciculations in the left intrinsic hand muscles and the distal forearm muscles simultaneously with the polyminimyoclonus. A T2-weighted cervical spine MRI scan in the flexed sagittal position revealed anterior displacement of the dura and an enlarged epidural space from C4-C7. At last follow-up 11 months later, the findings were unchanged. The EDX studies and cervical MRI findings were consistent with HD in both cases. The role of EDX studies and cervical spine flexion MRI in diagnosing HD and the correlation between polyminimyoclonus and fasciculations are highlighted.

## Introduction

Following Hirayama et al.'s report of 12 cases of juvenile muscular atrophy of the unilateral upper extremity in 1959 [1], Gourie-Devi et al. coined “monomelic amyotrophy” in 1984 to describe adolescent males with unilateral wasting and weakness of the upper extremity [2]. Hirayama disease (HD), also known as benign focal amyotrophy, juvenile monomelic amyotrophy, flexion myelopathy, and juvenile muscular atrophy of the distal upper extremity, is marked by the asymmetrical, progressive, atrophic weakness of the forearms and hands primarily involving the C7, C8, and T1 myotomes without sensory loss [3-8]. The topography of muscle wasting is often typical in sparing the brachioradialis with the forearm appearance of “oblique amyotrophy” [9]. Other features are tremulous movements of the fingers (“polyminimyoclonus”) and “cold paresis,” the tendency for muscle weakness to worsen on exposure to cold [3,7,8]. It is most common in males in the second and third decades. The condition has been more frequently reported in Asian countries, especially Japan and India [2-5,8,10,11], with only a few cases from the United States [12] and Europe [13,14]. HD has an insidious onset and self-limiting course [13], with gradual advancement for two to four years followed by arrest of progression [2,3,6-8,10,11,15].

A cervical spine MRI with neck flexion is the gold standard to diagnose HD, with characteristic findings of forward displacement of the posterior dural sac leading to anterior-posterior asymmetric flattening of the lower cervical cord resulting in ischemic and atrophic changes of the anterior horn cells and asymmetric atrophy of the lower cervical cord [3,4,6,8,10,13-15]. The anterior horn cells of the spinal cord often show shrinkage, necrosis, degeneration of large and small nerve cells, and gliosis [16]. An enhancing, crescent-shaped lesion in the posterior epidural space of the lower cervical canal may also be seen on flexion cervical spine MRI, representing a congested posterior internal vertebral venous plexus [3,15]. Hirayama postulated that the MRI changes were due to disproportional growth between the vertebral column and contents of the spinal canal, especially the dural sac, during the juvenile growth spurt [17]. The significantly higher number of males affected by HD may be attributed to the rapid height increase of males during puberty compared to females [7]. The juvenile-onset and male predominance in HD have been termed the disproportion hypothesis. Snake-eyes appearance (SEA) is marked by a symmetrical bilateral small high-signal-intensity lesion on an axial T2-weighted MRI [18]. The sequela is cystic necrosis at the junction of the central gray matter near the ventrolateral posterior column. The incidence of SEA in HD is 10.6% [18]. SEA reflects a poor prognosis and may be due to chronic compression and vascular insufficiency.

Electrodiagnostic (EDX) studies often demonstrate chronic denervation with or without acute denervation changes (fasciculations, positive sharp waves, and fibrillation potentials) mainly in the C7, C8, and T1 myotomes, discrete motor unit recruitment on volitional contraction with wide duration large amplitude polyphasic motor units, as well as decreased compound muscle action potentials (CMAP) amplitudes of the abductor digiti minimi (ADM), first dorsal interosseus (FDI), and abductor pollicis brevis (APB) muscles [3,5,10,13]. The sensory nerve conduction velocity (SCV) and sensory nerve action potential (SNAP) amplitudes are normal.

We present two cases of HD in teenage Caucasian males with unilateral hand muscle atrophy and polyminimyoclonus, small-amplitude jerky twitching in the fingers accompanying fasciculations in the intrinsic hand muscles suggestive of ventral horn cell involvement [19]. We discuss the differential diagnosis of HD, the importance of EDX studies and cervical spine flexion MRI in confirming the diagnosis of HD, and the correlation between fasciculations in the distal muscles and polyminimyoclonus.

## Case presentation

Case 1

History and Physical Examination

**Figure 1:**
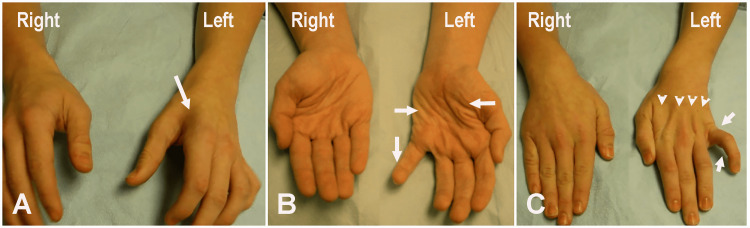
Marked wasting and weakness of the left intrinsic hand muscles (A) Case 1: marked wasting of the first dorsal interosseous muscle of the left hand (arrow). (B) Volar view showing inability to adduct the small finger of the left hand, resulting in the Wartenberg sign (vertical arrow). Note the wasting of the left thenar and hypothenar muscles (horizontal arrows). (C) Dorsal view demonstrating failure to adduct the left small finger, resulting in a positive Wartenberg sign (vertical arrow) and compensatory hyperextension at the metacarpophalangeal joint (oblique arrow). Note the wasting of the interosseous muscles on the left hand (arrowheads).

A 15-year-old male reported a six-month history of progressive weakness, wasting, and tremulous movements of the left hand without pain or paresthesia. He reported that he was unable to use a keyboard effectively or use his left thumb while playing video games. The patient denied symptoms in the right upper extremity or lower extremities. There was no history of trauma, neck pain, or vertigo; however, he did report syncopal episodes. A physical exam revealed marked wasting and weakness of the left intrinsic hand muscles (Figure 1A-1C). The extensors of the fingers were weak, while the wrist extensors were normal. Mild weakness of the flexor pollicis longus (FPL) and flexor digitorum profundus (FDP) was noted. Proximal muscles, including the biceps, brachioradialis, and deltoid, were normal. The biceps, triceps, and knee reflexes were symmetrical. The sensory examination was normal. Scoliosis of the thoracic spine leading to left shoulder elevation was observed.

EDX Studies

Stimulation of the left ulnar nerve with recording over the ADM showed markedly decreased amplitude of the CMAP, and the motor conduction velocity demonstrated focal slowing across the elbow. With recording electrodes over the FDI muscle, a drop in motor conduction velocity of 12 m/s across the left elbow was observed, and the amplitude of the CMAP was decreased. Sensory potentials over the left small finger as well as left superficial radial and medial antebrachial cutaneous nerves demonstrated normal latency and amplitude. The left median nerve showed prolongation of distal motor latency with low normal motor conduction velocity in the forearm segment. The amplitude of the CMAP over the APB was decreased. The sensory potentials showed a normal latency and amplitude without evidence of focal slowing across the left wrist in an “inching” study.

Needle electromyography (EMG) revealed 1-2 large polyphasic motor units in the left FDI and ADM muscles with fasciculations; the left APB, flexor carpi ulnaris (FCU), extensor indicis (EI), and triceps muscles showed decreased motor unit recruitment with large polyphasic units. The distribution of the EMG abnormalities suggested probable localization to the (1) lower trunk of the brachial plexus, (2) C8 and T1 nerve roots, or (3) anterior horn cells. The normal SNAPs of the ulnar and medial antebrachial cutaneous nerves indicated a lesion proximal to the lower trunk of the brachial plexus (preganglionic) at the C8 and T1 nerve roots or ventral horn cells. The left ulnar nerve neuropathy at the elbow with focal demyelination was considered an incidental finding. The slowing of the motor conduction in the left median nerve was explained based on the loss of fast-conducting motor axons and not from entrapment at the carpal tunnel in view of the normal “inching” study.

Radiological Findings, Surgical Intervention, and Follow-Up

**Figure 2:**
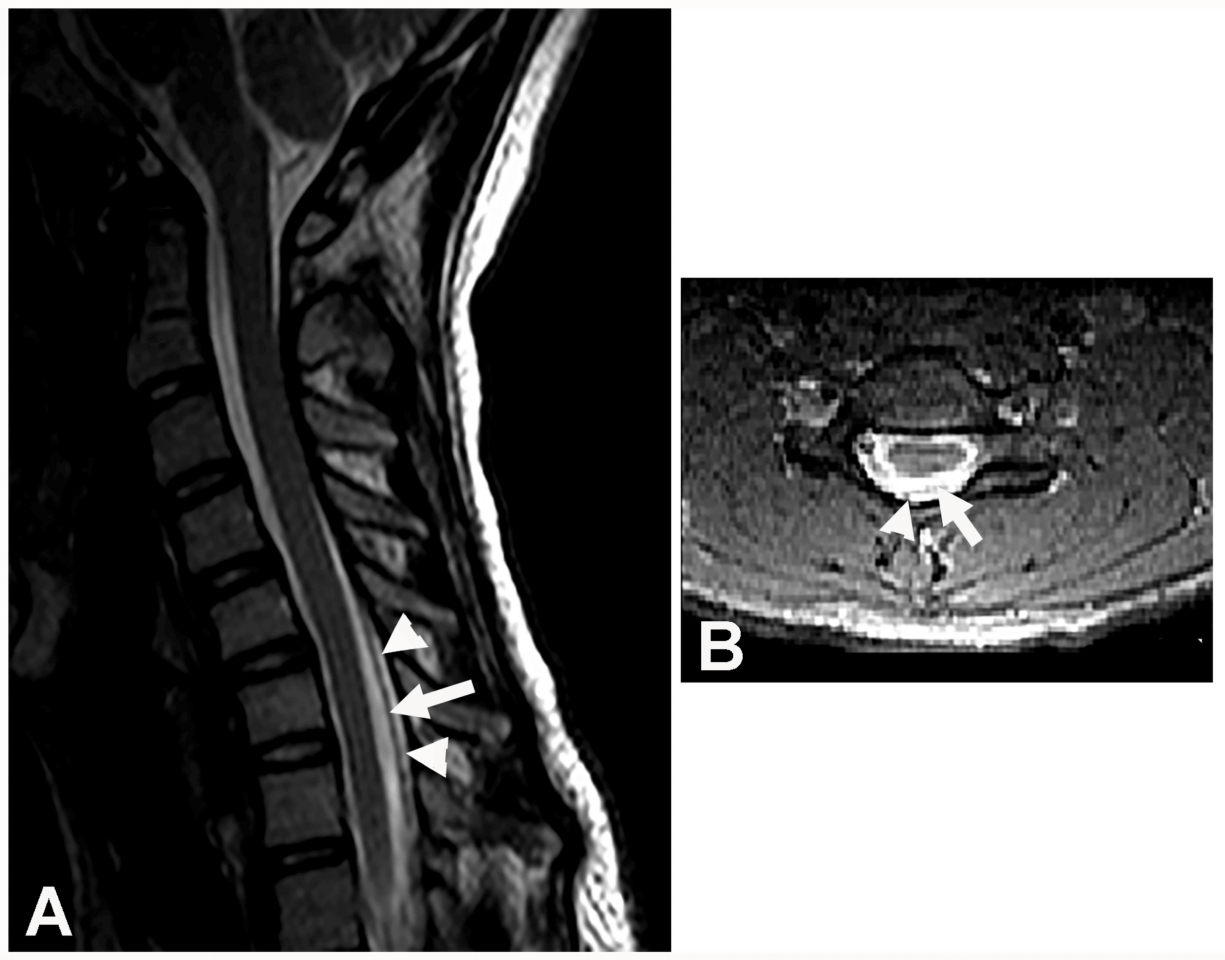
T2-weighted cervical spine MRI scan in the neutral position Case 1: T2-weighted cervical spine MRI scan in the neutral position. (A) Sagittal view demonstrating the normal position of the posterior dura (arrow), thin epidural space (arrowheads), and the normal position of the spinal cord. (B) Axial view of the cervical spine MRI scan of the lower cervical spine demonstrating the dura (arrow), normal amount of epidural fat (arrowhead), and slight atrophy of the left half of the spinal cord. MRI: magnetic resonance imaging

**Figure 3:**
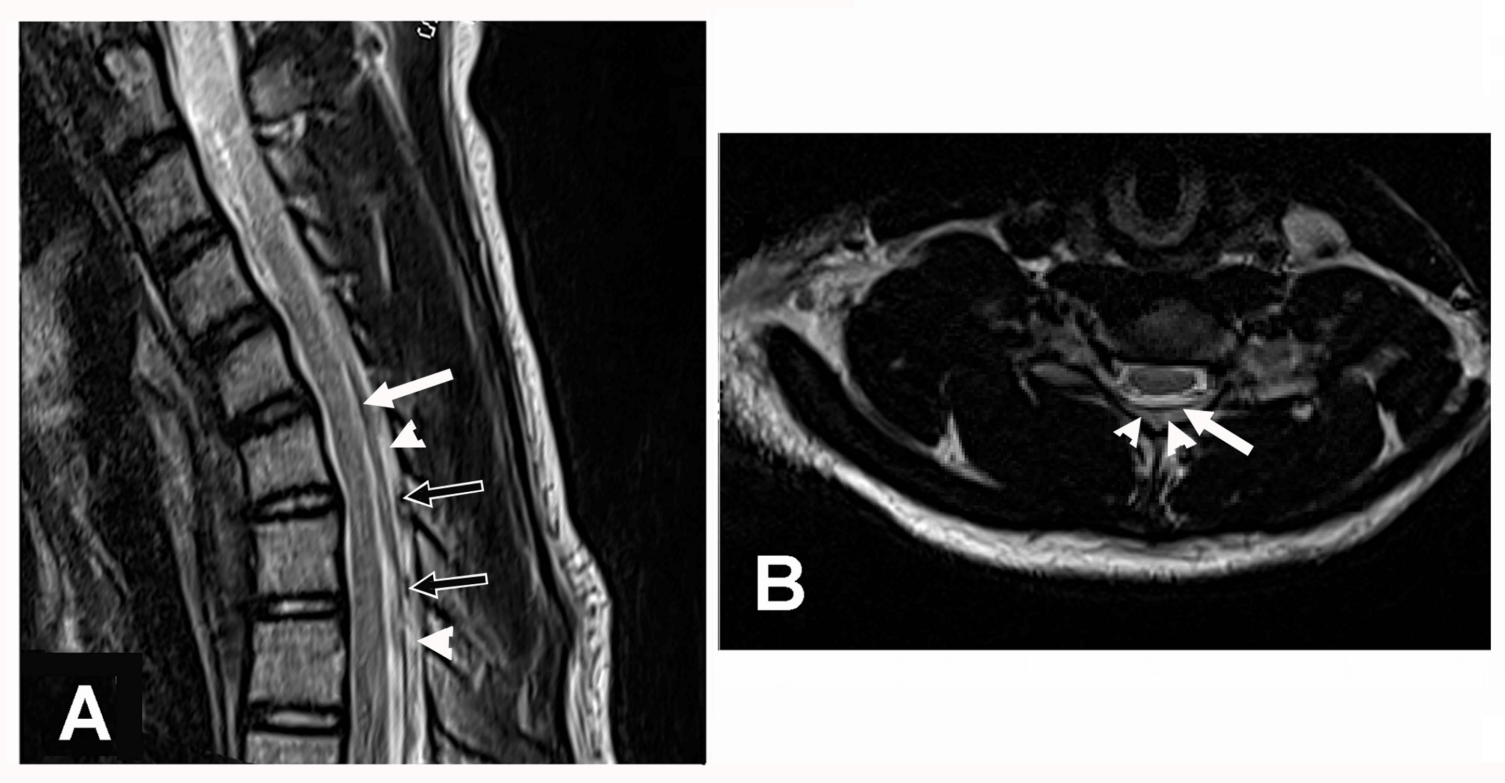
T2-weighted cervical spine MRI scan in the flexed position Case 1: T2-weighted cervical spine MRI scan in the flexed position. (A) Sagittal view demonstrating anterior displacement of the spinal cord, anterior displacement of the posterior dura (arrow), enlarged epidural veins (black arrows), and thickened epidural space (arrowheads). (B) Axial view showing slight atrophy of the left side of the spinal cord and the posterior margin of the thickened epidural space (arrowheads). The arrow signifies posterior dura. MRI: magnetic resonance imaging

A T2-weighted cervical spine MRI in the neutral position demonstrated the normal position of the posterior dura, thin epidural space, epidural veins, and the normal position of the spinal cord (Figure 2A-2B). A T2-weighted cervical spine MRI in the flexed position revealed anterior displacement of the spinal cord, anterior displacement of the posterior dura, enlarged epidural veins, and a thickened epidural space (Figure 3A-3B). These findings were consistent with the diagnosis of HD. The patient was evaluated by a pediatric neurosurgeon who did not recommend cervical spinal surgery. This decision was based on a thorough review of the literature on the rare condition of HD. The neurosurgeon determined that there was no surgical cure for HD and no absolute indication for any surgical intervention. The neurosurgeon did not recommend any treatment apart from wearing a cervical collar. The patient underwent a left cubital tunnel release at the elbow two months following the EDX studies, as it was unclear if the ulnar neuropathy may also be contributing to the muscle weakness. At last contact 11 years following symptom onset, the patient continued to experience profound weakness of the left hand without pain or numbness.

Case 2

History and Physical Examination

A 17-year-old male was found to have weakness and wasting of the muscles of the non-dominant left hand on a routine physical examination; the duration was unclear. He denied any neck pain and no symptoms in the right hand or lower extremities. There was no history of trauma. A physical exam revealed wasting of the left FDI, ADM, APB, and extensor pollicis longus (EPL) muscles, as well as the FDP and FCU muscles. Tremulous movements of the fingers were noted with irregular side-to-side and flexion/extension components at the metatarsophalangeal and interphalangeal joints (Videos 1-2). Fasciculations were noted in the FDI and the APB muscles. The sensory examination was normal.

**Video 1:** Polyminimyoclonus of the left hand Case 2: left hand showing polyminimyoclonus, which corresponds to fasciculations in the left hand and forearm muscles depicted in Video 3.

**Video 2:** Polyminimyoclonus of the left hand Case 2: left hand showing polyminimyoclonus, which corresponds to fasciculations in the left hand and forearm muscles depicted in Video 3.

EDX and Ultrasound Studies

Needle EMG demonstrated large amplitude, wide duration motor unit potentials in the left FDI, ADM, EI, APB, FPL, PT, and triceps muscles on voluntary contraction. Fasciculations were noted in the FDI, APB, and EI muscles. A comparison study showed a few large polyphasic units in the right FDI, EDI, and APB muscles. Nerve conduction studies showed normal amplitudes of sensory potentials in the ulnar, median, superficial radial, and medial antebrachial cutaneous nerves. The CMAP amplitude over the ADM and the APB muscles was decreased. Ultrasound study showed fasciculations in the APB, FDI, ADM, FDP, and EDC muscles (Videos 3-4). The EDX abnormalities suggested a lesion involving the C8, T1, and, to a lesser extent, C7 nerve roots or ventral horn cells.

**Video 3:** Ultrasound with probe in the left palm Case 2: ultrasound with probe in left palm. The lumbrical muscles on either side of the flexor tendon (vertical arrow) show frequent fasciculations.

**Video 4:** Ultrasound and needle EMG of the left thenar muscles Case 2: (A) ultrasound image showing fasciculations in left thenar muscles. (B) Needle EMG from left thenar muscles showing fasciculations. EMG: electromyography

Radiological Findings, Surgical Intervention, and Follow-Up

**Figure 4:**
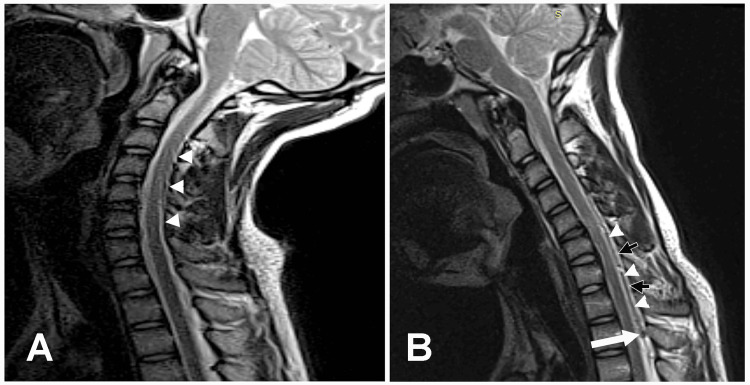
T2-weighted cervical spine MRI scan in the extended sagittal position Case 2: (A) T2-weighted cervical spine MRI scan in the extended sagittal position demonstrating the posterior dura (white arrowheads), thin epidural space from C4-C7, and no enlarged epidural veins. (B) T2-weighted cervical spine MRI scan in the flexed sagittal position revealing anterior displacement of the spinal cord and dura (white arrowheads), an enlarged epidural space from C4-C7 (black arrows), and an enlarged epidural vein (white arrow). MRI: magnetic resonance imaging

A T2-weighted cervical spine MRI scan in the extended sagittal position demonstrated the posterior position of the dura, thin epidural space from C4-C7, and no enlarged epidural veins (Figure 4A). A T2-weighted cervical spine MRI scan in the flexed sagittal position revealed anterior displacement of the dura, an enlarged epidural space from C4-C7, and an enlarged epidural vein (Figure 4B). These findings were consistent with the diagnosis of HD. At the last follow-up, 11 months later, no worsening of weakness was reported. The physical exam continued to reveal significant weakness of the left intrinsic hand muscles as well as EPL, APL, and EI muscles. Repeat needle EMG showed findings similar to the previous study. These findings demonstrated no disease progression since the first visit.

## Discussion

A number of clinical, EDX, and MRI characteristics point to the diagnosis of HD (Table 1) [2-8,10,11,13,15]. HD is a self-limiting condition, with most patients attaining a stable state three to five years after onset.

**Table 1:** Common characteristics of HD EDX: electrodiagnostic, MRI: magnetic resonance imaging, HD: Hirayama disease, CMAPs: compound muscle action potentials, ADM: abductor digiti minimi Table Credit: Lisa B.E. Shields, M.D. The table is the author's own creation and was designed based on the information in the following published articles [2-7,10,11,13,15].

Common characteristics of HD
Distal weakness and muscular atrophy in the hand and forearm sparing the brachioradialis (oblique atrophy)
Unilateral upper extremity involvement
Most common between the ages of 10-20 years
More frequent in males than females
More widespread in the Asian population
Insidious onset with gradual progression for 2-4 years followed by stabilization
No sensory or deep tendon reflex abnormalities
EDX studies often reveal chronic denervation changes in C7, C8, and T1 innervated muscles and decreased amplitude of CMAPs in the ADM; segmental neurogenic damages of anterior horn cells or anterior roots of the spinal nerves located in the lower cervical spinal cord
Cervical flexion MRI shows cervical cord compression with forward displacement of the posterior dura

**Figure 5:**
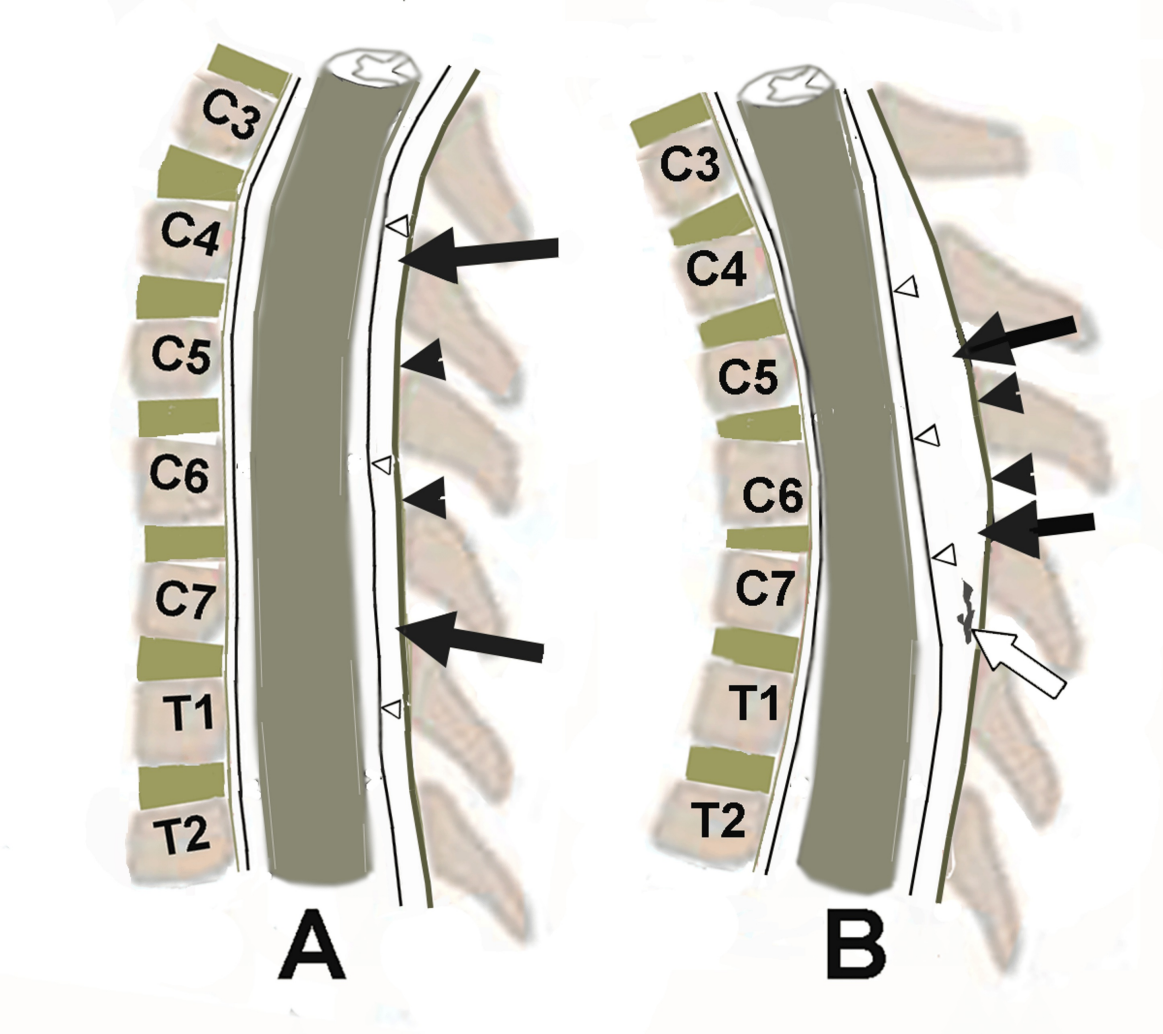
Schematic drawing depicting radiographic abnormalities in HD Schematic drawing highlighting the radiographic abnormalities in HD (sagittal view). (A) The neutral position of the cervical spine with the normal position of the spinal cord and posterior dura (triangles), a thin posterior epidural space (black arrows), and no epidural vein enlargement. (B) Flexed position of the cervical spine with anterior displacement of the spinal cord, anterior displacement of the posterior dura between C4-C7 (triangles), enlargement of the posterior epidural space (black arrows), and enlarged epidural veins (white arrow). Arrowheads delineate the ligamentum flavum. HD: Hirayama disease

A diagnostic feature is the forward displacement of the posterior dural sac during neck flexion, which was revealed by an MRI (Figure 5) [8].

Several conditions should be considered when a patient presents with asymmetric neurogenic atrophy of the hands, including neurogenic thoracic outlet syndrome, C8-T1 radiculopathy, syringomyelia, and ulnar nerve neuropathy in an “all ulnar” hand in addition to HD (Table 2).

**Table 2:** Differential diagnosis of our cases HD: Hirayama disease, SNAP: sensory nerve action potentials, MABC: medial antebrachial cutaneous, PIN: posterior interosseous nerve Table Credit: Vasudeva G. Iyer, M.D. The table is the author's own creation.

Differential diagnosis	Similarities to HD	Differences from HD
Neurogenic thoracic outlet syndrome	Chronic denervation in lower trunk distribution	Presence of pain and paresthesia and abnormal SNAP over ulnar and MABC nerves
C8-T1 radiculopathy	Denervation in C8-T1 distribution	Presence of pain and paresthesia
Syringomyelia	Chronic denervation diffusely distally	Presence of dissociated sensory loss
Ulnar nerve neuropathy in an “all ulnar” hand	Chronic denervation in all intrinsic hand muscles	Absence of denervation in the radial/PIN innervated C8, T1 muscles

The first three of these conditions demonstrate topography of denervation on EDX studies similar to HD due to involvement of the lower trunk in neurogenic thoracic outlet syndrome, C8-T1 nerve roots in C8-T1 radiculopathy, and ventral horn cells in syringomyelia. In the case of ulnar neuropathy in the all-ulnar hand, the C7-8 innervated radial nerve-innervated muscles will be spared, unlike C8-T1 root/ventral horn cell disorders. These disorders are often associated with pain and paresthesia (neurologic thoracic outlet syndrome and C8-T1 radiculopathy) or dissociated sensory loss (syringomyelia) unlike HD, where there are no sensory abnormalities. Disorders that mimic HD include amyotrophic lateral sclerosis (ALS), the distal form of spinal muscular atrophy, post-polio syndrome, multifocal motor neuropathy with conduction block, and toxic neuropathy [3,13].

EDX studies are a valuable tool in differentiating between the various conditions that mimic HD. In Guo et al.'s study of the clinical and neuroelectrophysiological features in 14 patients with HD, all patients reported muscular atrophy/weakness of the hand and/or distal forearm with atrophy primarily at the first interosseous muscle and thenar/hypothenar muscles [5]. All patients had denervation changes in the affected side in muscles innervated by the anterior horn cells at the lower cervical region (C7, C8, T1). Seven patients had bilateral upper extremity muscle neurogenic injury. The CMAPs of ulnar nerve-innervated muscles were lower than the reference value by >20% in 8, which exceeded the reduction in CMAPs of median nerve-innervated muscles. This finding was most likely due to the ADM muscle being the most commonly and severely affected by HD [5]. All patients had normal terminal motor latencies, SCVs, and SNAPs. Other findings that an EMG may uncover include the “reverse split hand syndrome” (decreased/absent CMAP amplitude in the ADM muscle while being preserved in the APB muscle), which may be observed in HD compared to the “split hand syndrome” (the combination of atrophy of thenar and the FDI muscles disproportionately wasted compared to the hypothenar muscles) often seen in ALS [10].

While a few studies have reported fasciculations of the affected muscles in patients with HD [4], correlation to polyminimyoclonus has rarely been reported in HD [6,11]. Polyminimyoclonus refers to involuntary, jerky, irregular, low amplitude, intermittent, and arrhythmic movements most frequently seen in the hands and fingers [19,20]. It is often observed when the hands are outstretched and the fingers extended. Polyminimyoclonus is associated with anterior horn cell disorders, including HD, and is most likely a clinical manifestation of exceedingly frequent fasciculations [20]. In Zhou et al.'s study of 192 patients with HD, tremors on finger extension were noted in 77.6% of patients [11]. In Vitale et al.'s study of eight patients with HD, three had a hand tremor [14]. In Meng et al.’s case report of a patient with HD, atrophy of the left FDI and tremulous movements of the left fingers with irregular jerky movements suggestive of polyminimyoclonus were observed [6]. These authors stress the importance of differentiating polyminimyoclonus from genuine tremor. We feel that the term “fasciculation-induced pseudotremor” is a more appropriate terminology for the tremulous movements of fingers seen in ventral horn disorders such as HD.

Early diagnosis and intervention in HD are associated with halting disease progression [15]. Patients with HD are usually treated with a soft cervical collar for three to four years, which aims to prevent neck flexion [12-14]. Other conservative measures include muscle-strengthening exercises and coordination of hand movements. Surgical intervention consists of either duraplasty or cervical spine decompression and fusion [8,10,13,15]. Case 1 in our study was treated with a soft cervical collar without undergoing cervical spine surgery. He continued to report marked weakness of the left hand 11 years after symptom onset. Case 2 had a shorter follow-up, and at 11 months after symptom onset, no worsening of weakness was reported. Repeat needle EMG showed no evidence of disease progression in the second case.

## Conclusions

HD is characterized by asymmetrical, progressive, self-limiting, and atrophic weakness of the forearms and hands mainly involving the C7, C8, and T1 myotomes in adolescent males. Both of our cases exhibited polyminimyoclonus, which appeared to be a consequence of fasciculations of the distal muscles such as the lumbrical and the interossei muscles as documented in the video displays. Even though less common in non-Asian countries, neurologists should still be cognizant of HD in juvenile males with asymmetric muscle wasting of the distal upper extremities, especially if polyminimyoclonus is also present. EDX studies coupled with MRI during neck flexion are useful in differentiating HD from its mimics.
